# Particle Characteristics’ Influence on FLASH Sintering of Potassium Sodium Niobate: A Relationship with Conduction Mechanisms

**DOI:** 10.3390/ma14051321

**Published:** 2021-03-09

**Authors:** Ricardo Serrazina, Camila Ribeiro, Maria Elisabete Costa, Luis Pereira, Paula M. Vilarinho, Ana M. O. R. Senos

**Affiliations:** 1 Department of Materials and Ceramic Engineering, Campus Santiago, CICECO—Aveiro Institute of Materials, University of Aveiro, 3810-193 Aveiro, Portugal; serrazina@ua.pt (R.S.); camila@ua.pt (C.R.); elisabete.costa@ua.pt (M.E.C.); 2 CENIMAT-I3N, Campus da Caparica, School of Science and Technology, FCT-NOVA, Universidade NOVA de Lisboa, 2829-516 Caparica, Portugal; lmnp@fct.unl.pt

**Keywords:** FLASH sintering, K_0.5_Na_0.5_NbO_3_, KNN, FLASH temperature, particle size, impurities, conduction mechanisms, ceramics, single crystals

## Abstract

The considerable decrease in temperature and time makes FLASH sintering a more sustainable alternative for materials processing. FLASH also becomes relevant if volatile elements are part of the material to be processed, as in alkali-based piezoelectrics like the promising lead-free K_0.5_Na_0.5_NbO_3_ (KNN). Due to the volatile nature of K and Na, KNN is difficult to process by conventional sintering. Although some studies have been undertaken, much remains to be understood to properly engineer the FLASH sintering process of KNN. In this work, the effect of FLASH temperature, T_F_, is studied as a function of the particle size and impurity content of KNN powders. Differences are demonstrated: while the particle size and impurity degree markedly influence T_F_, they do not significantly affect the densification and grain growth processes. The conductivity of KNN FLASH-sintered ceramics and KNN single crystals (SCs) is compared to elucidate the role of particles’ surface conduction. When particles’ surfaces are not present, as in the case of SCs, the FLASH process requires higher temperatures and conductivity values. These results have implications in understanding FLASH sintering towards a more sustainable processing of lead-free piezoelectrics.

## 1. Introduction

FLASH sintering is a powerful technique to sinter ceramics at low temperature in a very short period of time [1]. This occurs by the application of an electric field to a green compact. An increase in the specimen temperature promotes a raise in the conductivity, allowing sintering in seconds. The same is valid for a change in the operating atmosphere. [1,2]. The process is divided into three stages [3]: Stage I, the incubation, when the material is externally heated and the electric field promotes the creation and movement of electronic and/or ionic defects, stage II, when the FLASH event takes place at the FLASH temperature, T_F_, and heat generated by the Joule effect densifies the material at a very high rate, and stage III, or steady state, where the current is limited to avoid complete melting.

Recent reports revealed that, rather than applying an electric field to increase the current through the sample, a controlled current rate process produces denser and more uniform ceramics [4,5]. However, this approach makes it difficult to fully understand the role of the conductivity during stage I of FLASH sintering. In the present work, potassium sodium niobate, K_0.5_Na_0.5_NbO_3_ (KNN), a lead-free piezoelectric with technological relevance [6], was used to study the effect of green compact conductivity. Some studies show that the thermal cycle prior to the application of the electric field has a significant influence on the FLASH sintering process of KNN [7]. Additionally, the decrease in T_F_ was reported to occur with the increase in the applied electric field [8]. However, the impact of the initial powder purity and particle size in T_F_ and final densification of ceramics was not yet reported.

The strategies to decrease T_F_ have been associated with the increase in the overall conductivity of the green pellets, as for the case of YSZ (yttria stabilized zirconia), ZnO, or Al_2_O_3_, where both the decrease of particle size and the increase of impurity content decrease T_F_ [9,10,11]. However, the role of particles’ surface and their respective contact in semi-conductors (as ZnO) or ionic conductors (as YSZ) is not clear, as FLASH studies in single crystals of these materials reveal ambiguous results [12,13]. Besides, the conduction mechanisms and the influence of particle characteristics on FLASH sintering of KNN are not known. If KNN is to replace lead-based piezoceramics, its low-temperature processing is mandatory, not only for sustainability reasons but also because it is a technological challenge to sinter such alkali-based materials at high temperature [14,15]. In this work, the KNN FLASH process is studied using KNN powders with different purities and particle size distributions to establish the relation between T_F_ and the particle size and impurity content.

## 2. Experimental

K_0.5_Na_0.5_NbO_3_ (KNN) powders were prepared by a conventional solid-state route (Table 1). To achieve differences in powder purity, precursors with different purity were used. The final milling process was modified to promote changes on the particle size and morphology. In all cases, the precursors were dried before being mixed (in the stoichiometric amounts) in Teflon jars with yttria stabilized zirconia (YSZ) balls (ball:powder ratio of 7:1) and ethanol at 200 RPM, for 6 h. After mixing, the calcination was performed in alumina crucibles at 900 °C for 3 h, with 10 °C/min heating and cooling rates.

Table 1 shows the precursors used to produce two types of calcined KNN powders having different purity, which are labelled here as 99% KNN and 99.9% KNN, referring to the minimum precursor purity, respectively. After precursors’ mixing and calcination, KNN powders of the two batches (99% and 99.9%) were submitted to ball milling (BM) in ethanol, using Teflon jars and YSZ media (ball:powder ratio of 7:1), at 200 RPM, for 24 h. The ball-milled powders were designated (Table 1) as 99% BM and 99.9% BM powders, respectively. To achieve a decreased particle size, a batch of 99% KNN was submitted to attrition milling (AM) at high speed—in this case, Teflon jars and YSZ balls (ball:powder ratio of 20:1), in ethanol, were used at 700 RPM for 14 h—and the resulting powders were designated as 99% AM. A combination of both ball and attrition milling was also selected to decrease the particle size of 99.9% KNN. These powders were firstly ball-milled for 12 h, and then attrition-milled for 8 h, and designated as 99.9% BM+AM (Table 1).

The produced powders were analyzed by scanning electron microscopy (SEM, SU-70, 15 keV, Hitachi, Chiyoda, Japan) after being dispersed in ethanol with sonication. The particle size distribution (PSD) was accessed by particle laser diffraction (Coulter LS-200, Coulter Corporation, CA, USA) in water and used to estimate the average particle size, D50. The specific surface area (SSA) of the powders was determined by N_2_ adsorption in a Gemini 2.0 equipment (Micromeritics, Norcross, GA, USA), using the Brunauer, Emmett, Teller (BET) adsorption isotherm. The equivalent particle size (D_BET_) was calculated following Equation (1), where SF is the shape factor and *ρ*_t_ is the theoretical density of the material (4.5 g/cm^3^ for KNN):

(1) $$D_{\mathrm{BET}}=\frac{\mathrm{SF}}{\mathrm{SSA}\;\times \rho_{t}}$$

To access the crystalline structure of the produced powders, X-ray diffraction (XRD, XPERT-PRO PANalytical, Almelo, The Netherlands), with a copper X-ray source (Kα_1_ = 1.54060$\;\dot{A}$), was used. The chemical composition was determined by Inductively Coupled Plasma (ICP) mass spectrometry (Thermo Fisher Scientific, Waltham, MA, USA), by digesting the powders in a mixture of HNO_3_ and HF, using microwaves. A 100 mL solution volume was used to determine the content in K, Na, Nb, Al, and Zr.

Green compacts (ca. 15 × 5 × 2 mm^3^) were shaped by uniaxial (130 MPa, Carver, model C) and an additional isostatic pressing step (200 MPa, Autoclave Engineers). For isostatic pressing, compacts were vacuum-packed in rubber sleeves. After pressing, samples were characterized by SEM and the particle equivalent spherical diameter (D_eq._) was determined from particle cross-section area measurements—at least 500 particles were considered for the determination. The green density was geometrically determined.

FLASH sintering studies were performed in air using the produced KNN powders. An adapted dilatometer was used as previously reported [7], at a constant heating rate (C.H.R.) of 10 °C/min, an applied electric field of 300 V/cm, and a current limit of 20 mA/mm^2^. The furnace temperature and the respective sample size variation were recorded using an in-house developed software. The in-situ conductivity of KNN compacts was determined considering their initial dimensions and by measuring simultaneously the applied electric field and current flow in the system.

Sintered ceramics were analyzed by SEM, after being polished and etched (as reported in Reference [7]). The crystal structure of FLASH-sintered bodies was studied by powder XRD analysis. The final density of FLASH-sintered ceramics was determined geometrically (from mean values of geometrically measured volumes, using at least three different ceramics). To determine the relative density, the above reported value of 4.5 g/cm^3^ for the KNN theoretical density was considered.

High-density ceramics and single crystals (SCs) were produced to study the electrical conductivity of KNN polycrystalline ceramics and compared with that of SCs. Ceramics were obtained by FLASH sintering 99.9% BM powders in Isothermal Conditions (I.C.), as reported before [7], at an isothermal furnace temperature of 900 °C, with 300 V/cm applied after a 30 min dwell, a current limit of 20 mA/mm^2^, and a holding time of 60 s. K_0.5_Na_0.5_NbO_3_ SCs were produced by the high-temperature self-flux method, using 99% calcined KNN powders. The experimental details can be found in Reference [16]. To perform the direct current (DC) conductivity measurements of KNN bodies (ceramics and SCs), platinum electrodes were painted in ca. 1 mm thick ceramics and SC’s faces. After a drying step at 50 °C, the Pt electrodes were cured at 900 °C, for 1 h. The electrical conductivity was measured as a function of temperature, using a Keithley 2410 electrometer (Keithley Instruments, Solon, OH, USA). The specimens were heated at a constant rate of 10 °C/min, from 500 to ca. 950 °C, with an applied electric field of 1 V/cm. The activation energy for conduction (E_a_(σ)) of KNN bodies was determined based on an Arrhenius dependence, Equation (2), where σ is the conductivity, σ_0_ is the pre-exponential term, T is the temperature, and K_B_ is the Boltzmann constant:

(2) $$\ln\left(\sigma \right)=\ln\left(\sigma_{0}\right)-\;\frac{E_{a}\left(\sigma \right)}{K_{B}}\frac{1}{T}$$

## 3. Results and Discussion

### 3.1. Powders’ Characterization

**Figure 1:**
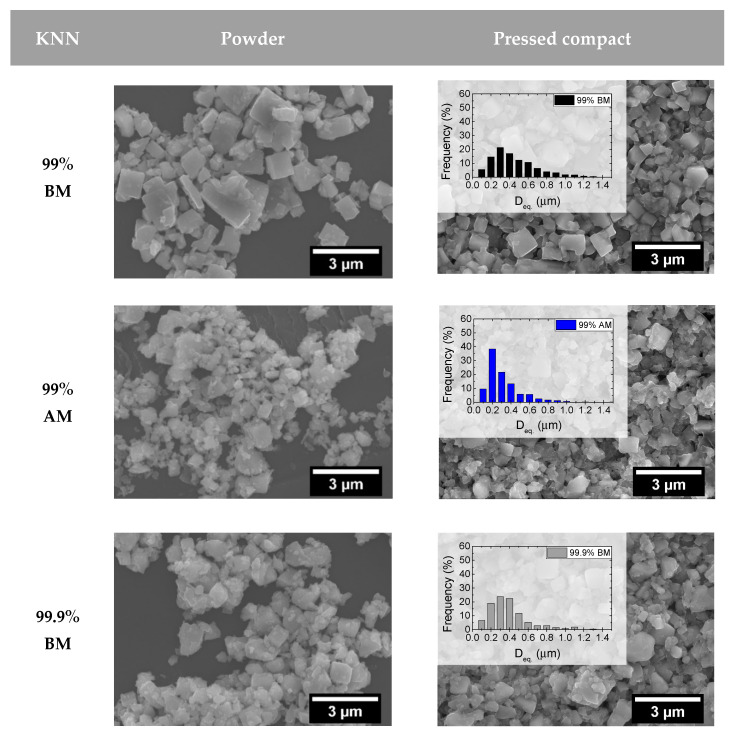

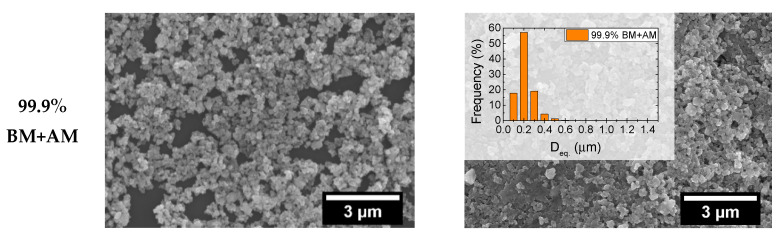
Scanning electron microscopy (SEM) micrographs of different K_0.5_Na_0.5_NbO_3_ (KNN) powders and pressed compacts with the respective particle size distribution, highlighting the dissimilarities in particle size.

Figure 1 shows the SEM analysis of loose powders (left) and respective green compacts (right). KNN particles are typically cuboid-shaped, which is clearly revealed for the produced powders, namely for the coarsest ones. The particle size distribution (PSD), estimated from particle area measurements, is overlapped with the micrographs in Figure 1. The data reveal that the two powders that were ball-milled (99% and 99.9%) present similar PSD. However, when attrition milling is employed (99% AM), the particle size is decreased, mostly at the expense of the size reduction of the largest particles. This effect is much more pronounced when a combination of ball and attrition milling is used (99.9% BM+AM).

Table 2 summarizes the obtained specific surface area (SSA) for each powder, together with the average particle size (D_BET_) determined from SSA, using Equation (1), considering a shape factor (SF) for cubes of 7.4. The mean particle sizes (D50) determined by PSD (D50-laser) and SEM (D50-SEM) are shown in Table 2, for comparison. The particle size D50 determined by the different techniques presents, as expected, different values, but reveals equivalent trends: the coarser powders are the ball-milled ones, with 99.9% BM (210–300 nm) being slightly finer than 99% BM (225–350 nm). On the other hand, when attrition milling is used alone (99% AM), particle size is decreased (171–210 nm). If ball and attrition milling (99.9% BM+AM) are combined, the particle size is markedly decreased (68–150 nm). In this case, the D50 analysis from SEM (150 nm) is bigger than both D50-laser and D_BET_ (86 and 68 nm, respectively). The same tendency was also observed for 99% AM. Difficulties in distinguishing the finer particles in SEM micrographs may have contributed to such observation. It should be noted that the particle size measurements by laser diffraction also presented some difficulties, due to minor KNN solubility in aqueous suspensions, and the agglomeration effects in fine powders. For these reasons, D_BET_ measurements are considered here as the most representative for particle size determination.

In summary, ball-milled powders (BM) are the most coarse, while the use of attrition milling (AM) leads to the decrease of the particle size. The combined use of ball and attrition milling (BM+AM) produces the finest powder.

In terms of impurity content, previous work has shown that aluminum is a possible contaminant in our KNN produced powders [14]. Furthermore, zirconium was also considered a possible contaminant from the erosion of YSZ balls during milling. Thus, ICP analysis was performed on the produced KNN powders (Table 3). Both K/Na and (K + Na)/Nb ratios are within the experimental error, in accordance with the K_0.5_Na_0.5_NbO_3_ stoichiometry. Al content in 99% powders is ~1 at%. On the other hand, the use of 99.9% pure precursors was effective to decrease Al contamination to residual values below 0.2 at%. Furthermore, the milling process did not promote relevant contamination from YSZ balls, and Zr content is ~0.1 at% in all the prepared powders.

**Figure 2:**
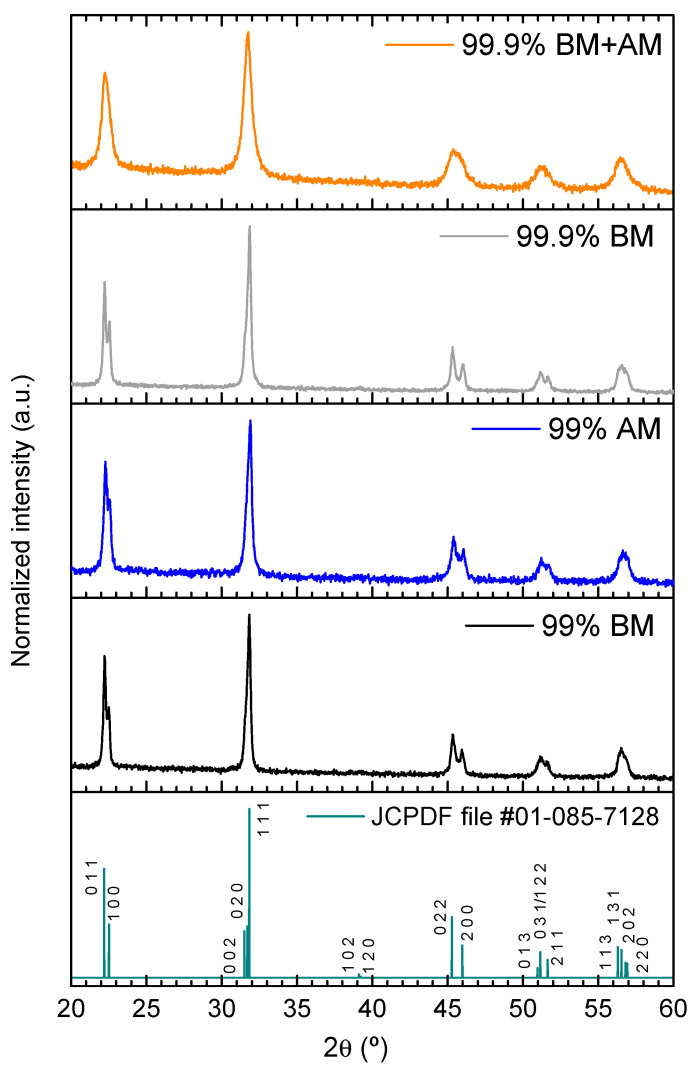
X-ray diffraction (XRD) patterns of produced KNN powders. JCPDF file #01-085-7128 of orthorhombic K_0.5_Na_0.5_NbO_3_ is included for comparison, and no secondary phases are identifiable.

X-ray diffraction (XRD) analysis does not reveal neither secondary phases nor relevant structural changes for any of the produced KNN powders (Figure 2). In fact, all XRD patterns agree with JCPDF file #01-085-7128, a monophasic orthorhombic K_0.5_Na_0.5_NbO_3_ phase. It is worth to notice that the 99.9% BM+AM powder XRD pattern presents less sharp maxima, which is another indication of its finer particle size.

### 3.2. FLASH Sintering Experiments

**Figure 3:**
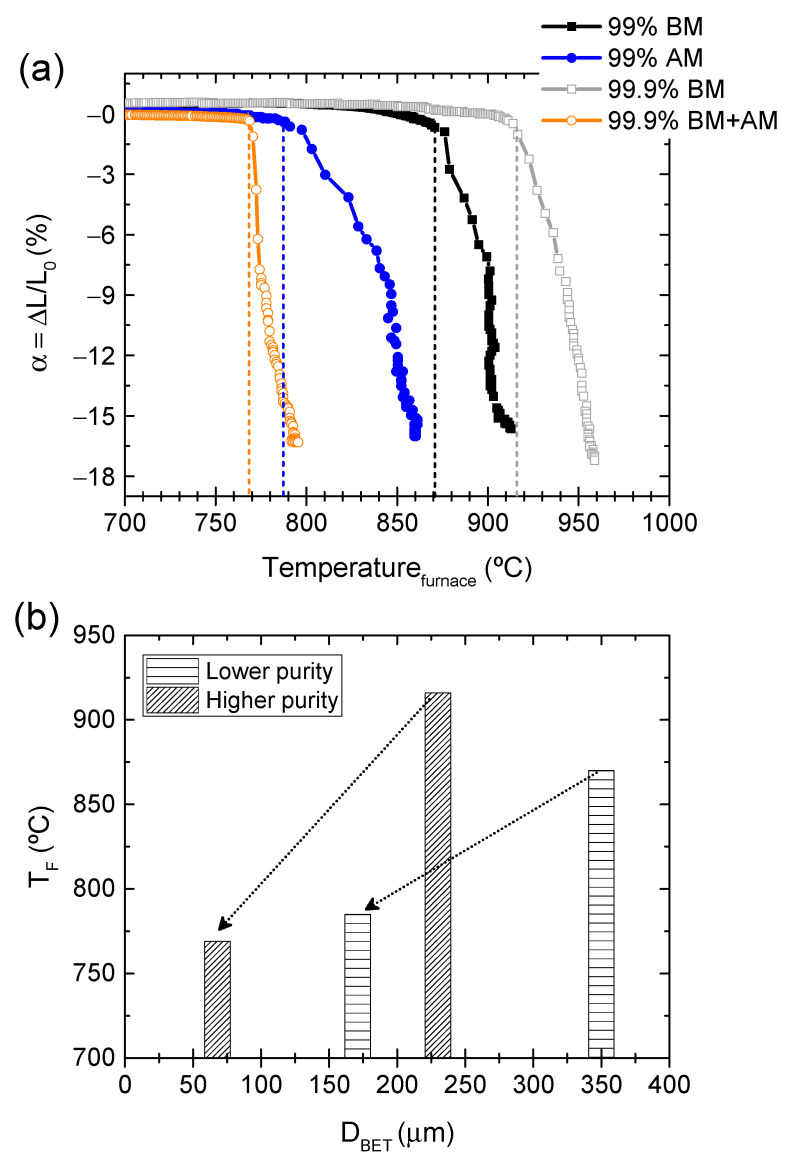
(**a**) relative displacement (α) as a function of furnace temperature during FLASH sintering of the produced powders (10 °C/min heating, 300 V/cm, 20 mA/mm^2^ current limit, 60 s holding time), and (**b**) schematic representation of T_F_ versus the particle size (D_BET_), revealing the tendency of T_F_ to decrease with particle size diminution and impurity content increase.

Dilatometric analysis was performed to study the FLASH sintering process of the produced KNN powders. Figure 3 presents the relative displacement (α) as a function of the furnace temperature during the sintering process. Data points were acquired with 1 s intervals.

The very fast shrinkage, typical of FLASH, is observable. The furnace temperature at which FLASH occurs (T_F_) is quite dependent on the powder chemical and morphological characteristics. From low to high T_F_ (dashed lines), the tendency is: 99.9% BM+AM (769 °C), followed by 99% AM (785 °C) and 99% BM (870 °C), with the highest temperature being for 99.9% BM (916 °C) (Figure 3a). These data are summarized in Table 4. Two relevant powder features that influence T_F_ must be considered: (i) particle size and (ii) impurity content. As clearly shown in Figure 3b, for the same impurity grade, the smaller the particles the lower the T_F_. In fact, the finest powder (99.9% BM+AM) can present even lower T_F_ than the other two impure powders with larger particle size, namely 99% AM. Moreover, T_F_ strongly decreases for similar particle sized powders (171 and 230 nm) with increasing Al-impurity content.

**Figure 4:**
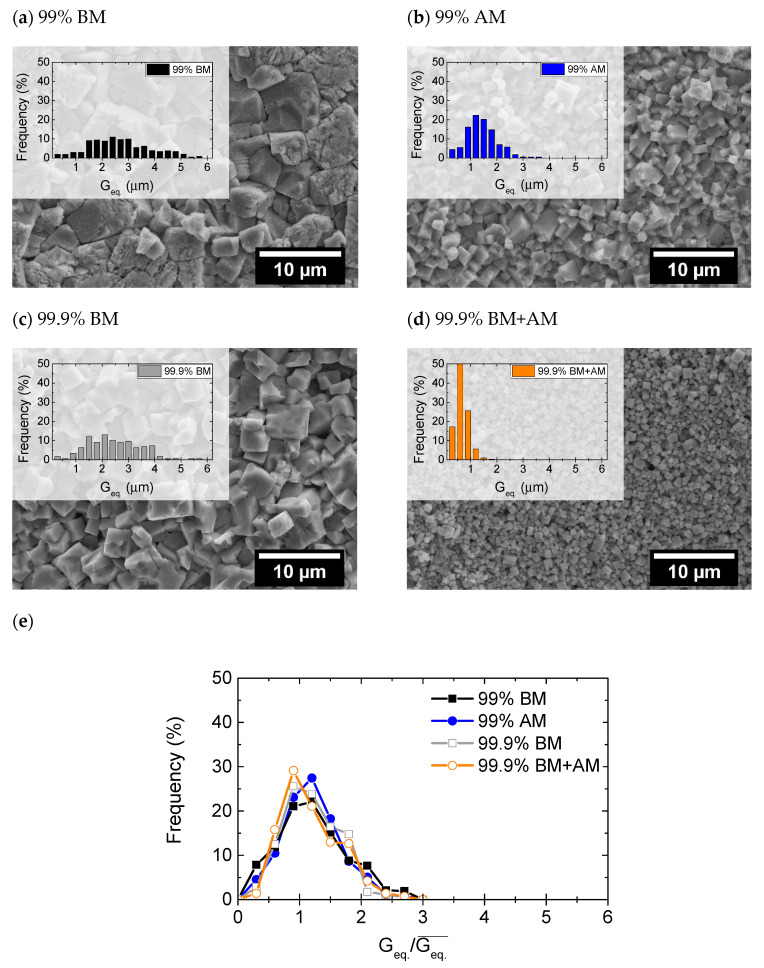
SEM micrographs of FLASH-sintered ceramics from (**a**) 99% BM, (**b**) 99% AM, (**c**) 99.9% BM, and (**d**) 99.9% BM+AM powders, with overlapped respective measured equivalent grain size (G_eq._) distribution, and (**e**) representation of the normalized (with the respective $\bar{G_{\mathrm{eq}.}}$) grain size distribution.

In contrast, the final shrinkage registered during the FLASH process is not significantly affected by the powder’s characteristics (−α = 16–17%). Figure 4 depicts the chemically etched micrographs of the ceramics, overlapped with the grain size distribution. Table 4 presents the green density, final density, and the respective mean grain size ($\bar{G_{\mathrm{eq}.}}$), estimated from the grain size distributions. In accordance with the final shrinkage (Figure 3a), and because the green density of compacts is equivalent (Table 4), there are no relevant dissimilarities in the final densification of FLASH-sintered ceramics. However, grain growth occurred during FLASH sintering of KNN and the final grain size is directly dependent on the starting particle size (Table 4 and Figure 4a–d). Nevertheless, Figure 4e reveals that the normalized grain size (by the respective average grain size of each distribution) is very similar, and such normalization results in a coincident distribution. Additionally, the coarser grains are not larger than ~2.7 $\bar{G_{\mathrm{eq}.}}$, which means that no abnormal grain growth occurred [17], regardless of the purity and PSD differences among the powders. The presence of impurities, usually segregated at grain boundaries, could result in dissimilar grain growth, if a classical grain boundary motion process was occurring [18]. This is not the case, and from the knowledge obtained so far, it is suggested that grain growth occurs by an Ostwald ripening mechanism. In this process, particles that are surrounded by a liquid [7,14] can grow in such a way that the finer ones dissolve and the solute precipitates on the coarser particles [19,20].

**Figure 5:**
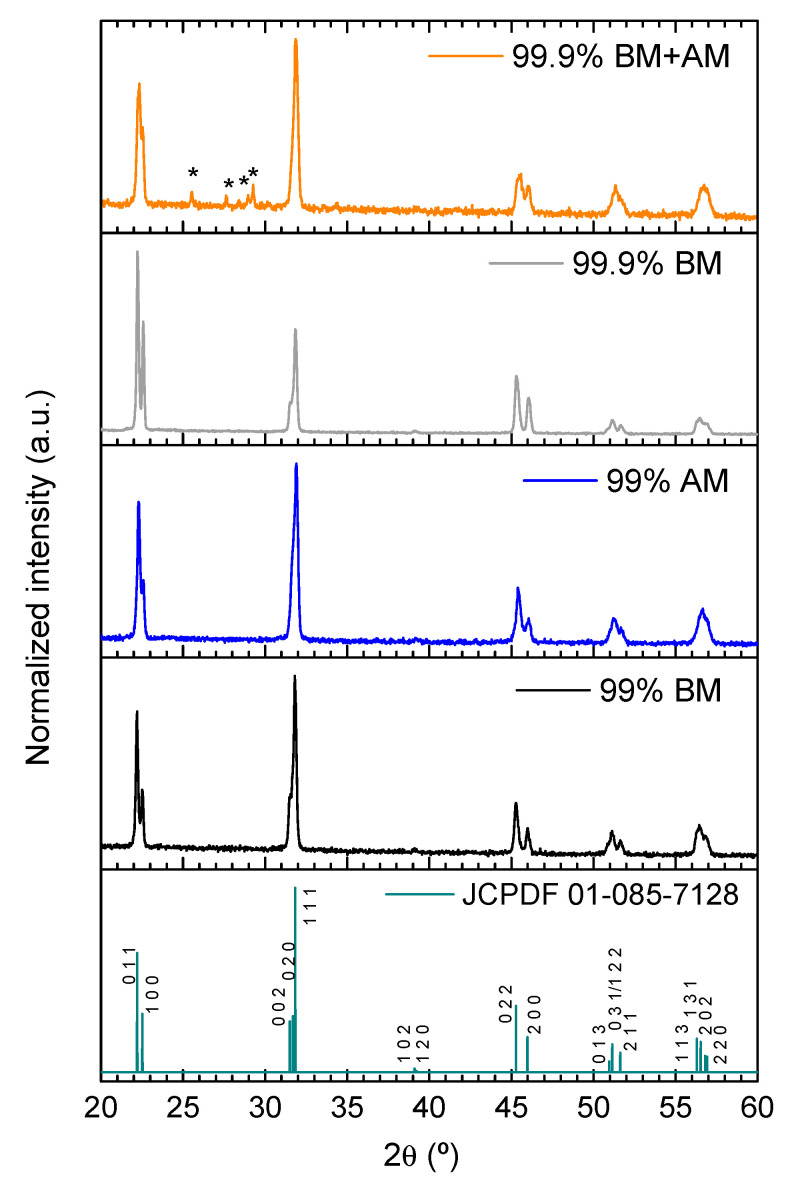
XRD patterns of FLASH-sintered ceramics. JCPDF file #01-085-7128 of orthorhombic K_0.5_Na_0.5_NbO_3_ is shown for comparison. Nb-rich secondary phases are identified with * for 99.9% BM+AM ceramic.

The XRD patterns of ground FLASH-sintered ceramics are presented in Figure 5. As described above, the influence of the particle morphology and impurities on the crystal structure of KNN powders (Figure 2) seems to be residual. However, Figure 5 reveals that this is not exactly the case for sintered ceramics. The XRD data indicates that no secondary phases are identified, except for 99.9% BM+AM, which presents Nb-rich secondary phases (K_1.3_Nb_5.8_ O_15_ and K_3_NbO_4_, with space groups 127 and 114, respectively). These are the finest powders, and because they are more reactive, the localized Joule heating might be high enough not only to promote the dissolution of finer particles, but also to locally vaporize alkali elements. This may explain the presence of Nb-rich secondary phases, which is deleterious from the application point of view [21]. In addition, a previously described inversion on the intensity of the first and second XRD reflections occurs for 99.9% BM [7], that might be associated with some degree of preferential orientation of KNN grains, as reported to occur in KNN thin films [22].

Therefore, it can be postulated that, while the particle size and impurity degree of KNN powders significantly influence the T_F_, the densification and grain growth processes are not appreciably affected, leading to final microstructures with the same densification and near equivalent grain growth (~7 times larger than the initial particle size). KNN phase remains stable in all the tested ceramics with the exception of the ones derived from the finest precursor powder, where a secondary Nb-rich phase was detected.

### 3.3. Mechanisms of Conduction during FLASH Sintering of KNN

**Figure 6:**
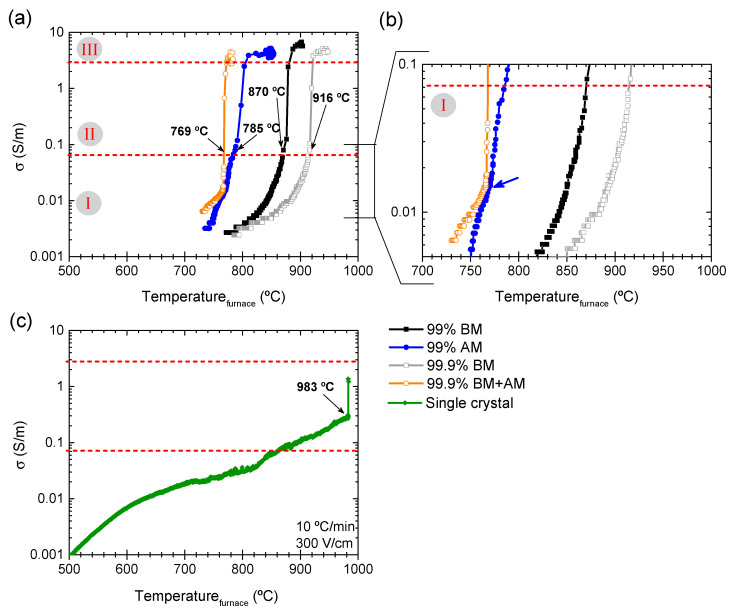
DC conductivity dependence (300 V/cm) versus furnace temperature for the (**a**) four different K_0.5_Na_0.5_NbO_3_ powders and (**c**) single crystal (SC). (**b**) shows a detail on the representation of (**a**). The effect of particle size/impurities is observed; additionally, the FLASH sintering of the SC is revealed.

To identify the reasons behind the significant dependence of T_F_ on the particle size and impurity content during the FLASH sintering of KNN, the electrical conductivity, σ, of green compacts was accessed (Figure 6a). These measurements were conducted under the same FLASH conditions previously used, i.e., 10 °C/min heating rate, 300 V/cm electric field, 20 mA/mm^2^ current limit, and 60 s holding time. In total agreement with Figure 3a, a typical in-situ FLASH sintering conductivity vs. temperature dependence is revealed for all the samples in Figure 6a. A first regime, stage I, has a slower increase of σ with temperature than in the second regime, stage II, where a very fast increase of σ is observed, and a current-limited one, stage III, where σ is nearly constant [3]. The transitions between these regimens are approximately identified with red horizontal dashed lines and respective designations in the figure. Despite the overall similar behavior, the curves presented in Figure 6a reveal the already discussed differences in T_F_ (see arrows and respective temperatures).

Besides T_F_, there are other powder-related features that change during the FLASH process. Figure 6b, a magnification of (a) for stage I, indicates that 99.9% KNN powders present a very similar stage I behavior, irrespective of the particle size (BM or BM+AM). It is characterized by a slow and steady increase of σ with temperature. However, for σ > 0.02 S/m, the finest powders (99.9% BM+AM) reveal a faster conductivity increase with temperature. On the contrary, the lowest purity graded KNN powders (99%) present stronger disruptions, especially identifiable for 99% AM, where at least two σ regimes are revealed: (i) a first one for σ < 0.015 S/m, and (ii) a second one for 0.015 σ < 0.07 S/m (see blue arrow in Figure 6b). This non-uniform increase in σ corresponds to the rather unstable shrinkage behavior visible in Figure 3a.

During stage II (FLASH event) and III (steady stage), there are further differences to highlight (Figure 6a). Although stage II seems very similar among the tested KNN powders, 99.9% BM+AM presents a significantly faster process than the others. Regarding stage III, fine powders (both 99% and 99.9%) display more disperse values of maximum conductivity, while the behavior of coarse ones is more constant.

As previously discussed, as the size decreases, the number of particle-to-particle contacts per unit of volume (contacts density) increases. By considering that a regular body centered cubic (BCC) arrangement of spheres, with a packing factor of 68%, close to the determined *ρ*_green_ of KNN pellets, would be representative of particle packing in a green KNN body, an average coordination number of 8 can be assumed. In this case, for a particle size decrease from 300 to 100 nm, an increase of about ~100 times in the density of particle contacts can be estimated. Thus, the overall conductivity of a green compact is expected to be significantly augmented as the number of contact points is increased, leading to the decrease of T_F_ [1,23]. Besides, the contribution of surface conduction for the overall conduction is enhanced in relation to that of bulk.

Additionally, as the density of the particle contacts is increased, the number of local electrical discharges is augmented during stage III, raising the dispersion of measured σ values in that stage. In parallel, if the powder impurity degree is increased, the concentration of conductive defects, as interstitials, vacancies, electrons, and holes, is also enhanced, decreasing T_F_. This effect was also described for impure and MgO-doped alumina [9,10].

In face of the proposed explanation for the T_F_ dependence on the particle size, regarding the relation between particle-contact density and impurity presence contribution for the overall compact conductivity, the role of particle surfaces and contacts for that process must be clarified. In fact, the previously proposed FLASH sintering mechanism of KNN states that the current flows through particle surfaces, in a network of current pathways uniformly distributed in the compact [7,14,24]. To truly validate this mechanism, the conductivity of surface versus bulk KNN must be considered. KNN single crystals (SCs) were then used for this purpose.

Figure 6c presents the conductivity behavior of KNN SCs under similar conditions to those used in FLASH sintering of compacts (Figure 3a and Figure 6a). As a first note, KNN SCs do FLASH (black arrow in Figure 6c). However, the FLASH process occurs at higher temperature (T_F_ = 983 °C) and conductivity values (σ = 0.3 S/m) than those observed for powder compacts. This observation agrees with the data reported for ZnO [13] and suggests that the presence of particle contacts allows T_F_ to be decreased as the density of contacts is augmented. Furthermore, SCs exhibit an “activated-state”, i.e., a jump in conductivity for values higher than 1 S/m (stage III), although the maximum conductivity reached during this stage is lower than that of compacts (σ = 1.4 S/m).

Zhang and co-workers [13] proposed that, in the case of ZnO, enhanced electronic conduction through the particles’ surfaces occurs in polycrystals, while it does not occur in SCs. This was the reason pointed out for the decrease in T_F_, when comparing poly with single crystals. However, in the case of 8 mol% cubic YSZ, the opposite was reported: SCs presented lower T_F_ than the respective powder specimens [12]. It was suggested that the surface conductivity in YSZ is lower than the bulk one. However, a detailed explanation for the observation was not provided, as the authors were focused on studying the power dissipation effect, which is very similar in powders and SCs [12].

The reported measurements of in-situ conductivity of single and polycrystalline samples fall in debatable accuracy issues, for two main reasons: the dissimilar scale of single crystals (mm) and polycrystalline pellets (cm) and the densification degree of each: fully dense for SCs, and 35% to 37% porosity in compacts. Issues with scaling of the applied potential for a constant electric field in different sized samples are known [25], as well as the detrimental effects of air (in porous compacts) for conductivity measurements.

Therefore, in this work, due to the interest in understanding the role of particle interfaces in T_F_ and the respective FLASH sintering process, a modified method is proposed to study the conduction mechanisms of KNN powders and SCs. A low-magnitude DC electric field (1 V/cm) was used in similar sized, previously densified polycrystalline ceramics and SCs, thus reducing the influence of external factors such as the scaled electric potential and porosity. 99.9% BM powders were used to produce highly dense polycrystalline FLASH-sintered KNN ceramics in Isothermal Conditions (I.C.), as previously reported [7].

**Figure 7:**
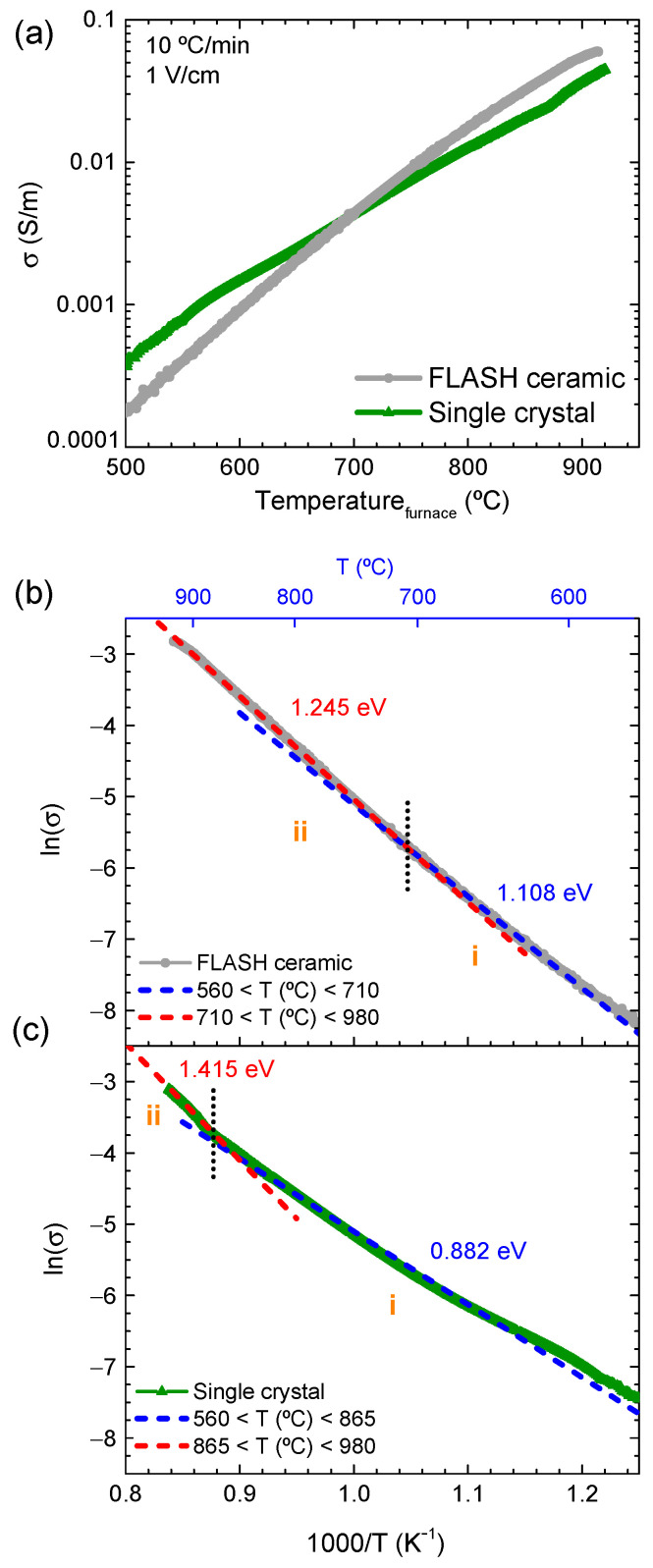
(**a**) DC conductivity vs. furnace temperature and respective Arrhenius plot of (**b**) KNN FLASH-sintered ceramics and (**c**) single crystal, with applied 1 V/cm electric field. The mechanisms of conduction are depicted by the respective activation energies, calculated for specific temperature regimes (i, and ii).

The dependence of DC conductivity versus the furnace temperature for FLASH-sintered ceramics and SCs is represented in Figure 7a. FLASH does not occur under 1 V/cm, either in ceramics or in SCs. The electric field here is significantly lower than the one previously used (300 V/cm). The DC conduction processes in KNN ceramics and SCs is thermally activated, as σ increases with temperature. Furthermore, for temperatures between 500 and ~710 °C, SCs present higher conductivity than the equivalent ceramics, whereas the opposite happens for T > 710 °C. From the best of our knowledge, the high-temperature (T > 500 °C) DC conductivity of KNN ceramics and SCs is reported here for the first time. Alternating current (AC) measurements were previously described in our group by Rafiq et al., for KNN ceramics and SCs, however, for T < 500 °C [16]. In the present work, the low limit of the DC representation (Figure 7a) is 500 °C, because at lower temperature, the samples are highly resistive, thus leading to noisy, not representative measurements. Even though a similar trend between AC and DC conductivity measurements is revealed for the temperature range 500 to 710 °C (Figure 7a), i.e., the conductivity of KNN ceramics is lower than that of SCs. To further understand the conduction process of KNN, the activation energy for conduction (E_a_(σ)) must be estimated.

The Arrhenius representation of the DC conductivity (1 V/cm) for FLASH-sintered ceramics and SC is shown in Figure 7b,c, respectively. E_a_(σ) is calculated for the two temperature regimes, identified in the figures by i and ii (not directly related with FLASH stages I, II, or III). The linear regressions (and the respective E_a_(σ) estimations) are represented with dashed lines, and E_a_(σ) values and respective temperature regimes are summarized in Table 5. Regimes i and ii occur at different temperatures for ceramics and SC and are differentiated by a low activation energy (E_a_(σ) < 1.2 eV) for the first, and a high activation energy (E_a_(σ) > 1.2 eV) for the second. The transition between each one occurs at T ~ 710 °C for ceramics, and at T ~ 865 °C for SCs.

Activation energies for conduction in ferroelectric perovskites reported between 0.4 and 1.2 eV are associated with charge transport by oxygen defects, namely, ionized oxygen vacancies [26,27], which was previously confirmed in KNN ceramics and SCs [16]. On the other hand, E_a_(σ) > 1.2 eV have been associated with ionic-based conduction mechanisms [16]. These activation energies are referred to AC studies. While DC conduction behavior might be different from the AC one, previous work on vanadium-alumina ceramics revealed that similar values of E_a_(σ) may be estimated from both DC and AC conductivity measurements [28]. Therefore, it is assumed here that conducting species and respective mechanisms operating at high-temperature DC conduction of KNN, namely conducting electrons from ionized oxygen vacancies, are comparable to those previously reported in AC experiments [16]. Additionally, the temperature regimes at which each mechanism is activated can be dependent on the signal. While the contribution of alkaline ions for the conduction (which was previously reported for SCs in AC studies [16]) is not ruled out in the present DC studies at the highest temperatures (where Ea(σ) > 1.2 eV), their representation might be conditioned by polarization effects at the electrodes [29].

Admitting that a direct link between reported AC-E_a_(σ) [16] and estimated DC-E_a_(σ) (this work) are comparable, the data of Figure 7b,c and Table 5 indicate that, in the case of SCs, while for T < 865 °C an oxygen vacancy-based charge transport phenomena occurs (0.4 < E_a_(σ) < 1.2 eV is valid), for T > 865 °C, ionic conductivity might be a contributing mechanism [16]. For ceramics, a similar behavior is identified, however, the considered ionic conductivity is revealed for T > 710 °C, as E_a_(σ) slightly overcomes the 1.2 eV threshold. We assume that the expression of ionic conductivity in both cases can be delayed to higher temperatures, as a consequence of electrode polarization [29]. To undoubtedly establish the alkaline ionic contribution for conduction in KNN SCs and ceramics during DC excitation, further studies shall be conducted. Here, we assume that the condition of E_a_(σ) > 1.2 eV is valid for such mechanism.

An additional fact is that ceramics are composed of grains surrounded by grain boundaries (GBs). Hence, the overall DC conductivity of ceramics is limited by that of GBs, as they form a network of pathways surrounding the grains. In AC measurements [16,30], the conductivity of bulk (grains) and grain boundaries is frequently differentiated, as the conducting species (ions, defects, electrons, polarons, or others) are sensitive to the change in the frequency [27]. While GBs are associated with chemical and structural discontinuities or disorders [31], with higher concentration in electronic defects and impurities, their low-temperature conductivity is typically lower than that of grains [32]. This occurs because such defects and impurities are localized, creating Schottky barriers for the charge carrier transport. In fact, Tkach et al. have shown, through impedance spectroscopy, that in SrTiO_3_ (at T < 600 °C), the conductivity of GBs is, if not lower, similar to that of grains [30]. These facts may explain the lower DC conductivity of KNN ceramics at low temperature (T < 710 °C) in comparison with that of SCs (Figure 7a). Furthermore, it may also explain the higher activation energy for conduction observed for ceramics, in comparison with SCs, during regime i (Table 5).

However, literature reports that the grain boundary space charge layer width can reach ~100 nm [32], and, in semiconductors, for small grain sizes, the width of space charges can be high enough to allow GB conduction, as the grains became depleted [33]. Thus, the contribution of GBs for conduction may be increased by the raise in the volume ratio of GB/grain. Nonetheless, in the present study, GB conduction is only revealed at high temperature (T > 710 °C), with the conductivity of ceramics overcoming that of SCs. At such point (T ~ 710 °C), the temperature is high enough to allow ionic mobility at the GBs of ceramics. Thus, the overall ceramic conductivity is increased, with a correspondent increase in the activation energy [16]. In SCs, the ionic conduction (from bulk KNN) is only revealed at higher temperature (T > 865 °C).

The proposed mechanism for GB-based conduction in ceramics is in good agreement with the observations of T_F_ decrease with the respective particle size decrease, as the volume ratio of surface/bulk is increased. Furthermore, the fact that the FLASH sintering of KNN compacts occurred at lower temperature than that of SCs is also explained, in accordance with previous work in ZnO [13]. A direct link between FLASH process and ionic conduction in KNN was found.

In summary, it is suggested that, when the temperature is high enough, the ions (and other conducting species as vacancies) at the ceramics’ GBs are thermally activated and the conduction is increased, overcoming the bulk conductivity of SCs. Being a surface phenomenon, as conductive as the surface is (high content in impurities) or the higher its volume ratio with respect to the bulk (small particle size), the lower the temperature needed to promote a long-range conduction process. When the electric field is high enough, that temperature is T_F_. Additionally, our previously reported and proposed particle surface-based KNN FLASH sintering mechanism [7,14] agrees with the findings presented here.

## 4. Conclusions

The dependence of FLASH temperature, T_F_, on particle size and impurity content of KNN powders was studied. It is concluded that while the densification and grain growth processes of FLASH-sintered KNN ceramics are not affected by the particle size and impurity content of precursor powders, T_F_ is. This is due to the raise in the density of particle contacts with the decrease in particle size. We proposed that the DC electrical conduction of ceramics is dominated by the grain boundaries, which is directly linked with the development of the FLASH sintering as a particle surface conductivity process. Thus, the smaller the particle size, and the greater the concentration of impurities, the lower the T_F_. This work shows that the density of particle contacts and surface-based conduction mechanisms dictate the FLASH temperature.

## Figures and Tables

**Table 1:** Precursors and experimental details on powder preparation.

	Precursors			Final Milling Step			
KNN	K	Na	Nb	Process	Medium	Speed (RPM)	Time (h)
**99% BM**	K_2_CO_3_ Merck 99.0%	Na_2_CO_3_ Chempur 99.5%	Nb_2_O_5_ Alfa Aesar 99.9%	Ball milling	Ethanol + YSZ balls	200	24
**99% AM**				Attrition milling		700	14
**99.9% BM**	K_2_CO_3_ Sigma-Aldrich 99.99%	Na_2_CO_3_ Sigma-Aldrich 99.999%		Ball milling		200	24
**99.9% BM+AM**				Ball + attrition milling		200 + 700	12 + 8

Note: Merck: Darmstadt, Germany; Chempur: Piekary Śląskie, Poland; Alfa Aesar: Kandel, Germany; Sigma-Aldrich: Saint Louis, MO, USA.

**Table 2:** KNN powders’ physical characteristics. Specific surface area (SSA) and respective equivalent particle diameter (D_BET_), and mean particle size from laser diffraction (D50-laser) and SEM images (D50-SEM).

KNN	SSA (m^2^/g)	D_BET_ (nm)	D50-Laser (nm)	D50-SEM (nm)
**99% BM**	4.75	350	235	350
**99% AM**	9.61	171	204	210
**99.9% BM**	7.22	230	210	300
**99.9% BM+AM**	24.1	68	86	150

**Table 3:** Inductively Coupled Plasma (ICP) mass spectroscopy analysis of KNN powders: the alkali ratios and the presence of impurities related with the precursor’s purity grade.

Powder	K/Na	(K + Na)/Nb	Al (at%)	Zr (at%)
**99% BM**	1.0 ± 0.1	1.1 ± 0.1	1.1 ± 0.1	0.10 ± 0.01
**99% AM**				
**99.9% BM**			0.14 ± 0.01	
**99.9% BM+AM**				

**Table 4:** FLASH temperature (T_F_), relative densities—green (*ρ*_green_) and after sintering (*ρ*_sint_)—and mean grain size ($\bar{G_{\mathrm{eq}.}}$ ).

KNN Powder	Compact		FLASH-Sintered Ceramic	
	*ρ*_green_ (%)	T_F_ (°C)	*ρ*_sint_ (%)	$\bar{G_{eq.}}\;(\mu m)$
**99% BM**	63 ± 2	870	89 ± 2	2.4
**99% AM**	65 ± 2	785	88 ± 3	1.2
**99.9% BM**	65 ± 1	916	89 ± 1	2.2
**99.9% BM+AM**	63 ± 2	769	89 ± 1	0.5

**Table 5:** Activation energies for the DC conductivity of KNN ceramics and single crystal (SC).

Temperature Range (°C)	E_a_(σ) (eV)	
	Ceramic	SC
560–710	1.108 ± 0.002	0.882 ± 0.002
710–865	1.245 ± 0.001	
865–980		1.415 ± 0.009

## Data Availability

Not applicable.
